# Diagnostic Value of sST2 in Cardiovascular Diseases: A Systematic Review and Meta-Analysis

**DOI:** 10.3389/fcvm.2021.697837

**Published:** 2021-07-23

**Authors:** Tianyi Zhang, Chengyang Xu, Rui Zhao, Zhipeng Cao

**Affiliations:** ^1^Department of Forensic Pathology, School of Forensic Medicine, China Medical University, Shenyang, China; ^2^Department of Forensic Pathophysiology, School of Forensic Medicine, China Medical University, Shenyang, China; ^3^Department of Forensic Medicine, School of Basic Medical Sciences, Fudan University, Shanghai, China

**Keywords:** sST2, meta-analysis, cardiovascular diseases, biomarker, heart failure

## Abstract

Biomarkers such as B-type natriuretic peptide (BNP), N-terminal pro-BNP (NT-proBNP), cardiac troponin (cTn), and CK-MB contribute significantly to the diagnosis of cardiovascular disease (CVD). Recent studies have demonstrated that suppression of tumorigenicity 2 (ST2) is associated with CVD, but a meta-analysis of ST2 levels in different CVDs has yet to be conducted. Therefore, the present study aimed to investigate soluble ST2 (sST2) levels in patients with ischemic heart disease (IHD), myocardial infarction (MI), and heart failure (HF). A total of 1,425 studies were searched across four databases, of which 16 studies were included in the meta-analysis. The Newcastle–Ottawa Quality Assessment Scale (NOS) values of all 16 studies were ≥7. The meta-analysis results indicated that the sST2 level was not correlated with IHD (standard mean difference [SMD] = 0.58, 95% confidence interval [95% CI] = 0.00 to 1.16, *p* = 0.05) or MI (weighted mean difference [WMD] = 0.17, 95% CI = −0.22 to 0.55, *p* = 0.40) but was significantly associated with HF (WMD = 0.21, 95% CI = 0.04 to 0.38, *p* = 0.02; *I*^2^ = 99%, *p* < 0.00001). sST2 levels did not differ significantly between patients with IHD or MI and healthy individuals; however, we believe that ST2 could be used as an auxiliary diagnostic biomarker of HF.

## Introduction

Cardiovascular disease (CVD) includes all pathologies of the heart or circulatory system, such as coronary artery disease (CAD), myocardial infarction (MI), heart failure (HF), and peripheral vascular disease. In 2019, the prevalence of CVD reached 523 million cases, resulting in 18.6 million deaths worldwide, and the number is still increasing, seriously affecting patient quality of life (1–4). In addition to successful rescue, early diagnosis and effective monitoring for patients with critical illness after disease onset are also needed.

Clinical biomarkers such as cardiac troponin (cTn), B-type natriuretic peptide (BNP), and N-terminal pro-BNP (NT-proBNP) are widely used in the diagnosis of MI and HF (5–7). Recent studies have demonstrated that suppression of tumorigenicity 2 (ST2) is also associated with CVD (8). ST2 is a member of the interleukin (IL)-1 receptor family and was first described in relation to inflammatory and autoimmune diseases in 1989 (9, 10). ST2 and its ligand interaction of IL-33 are involved in the immune response, and soluble ST2 (sST2) levels in plasma are elevated in patients with asthma, septic shock, trauma, and systemic lupus erythematosus (11). Blood sST2 levels are increased not only in inflammatory diseases but also in various heart diseases and are considered a valuable prognostic marker for CVD (12).

**Figure 1:**
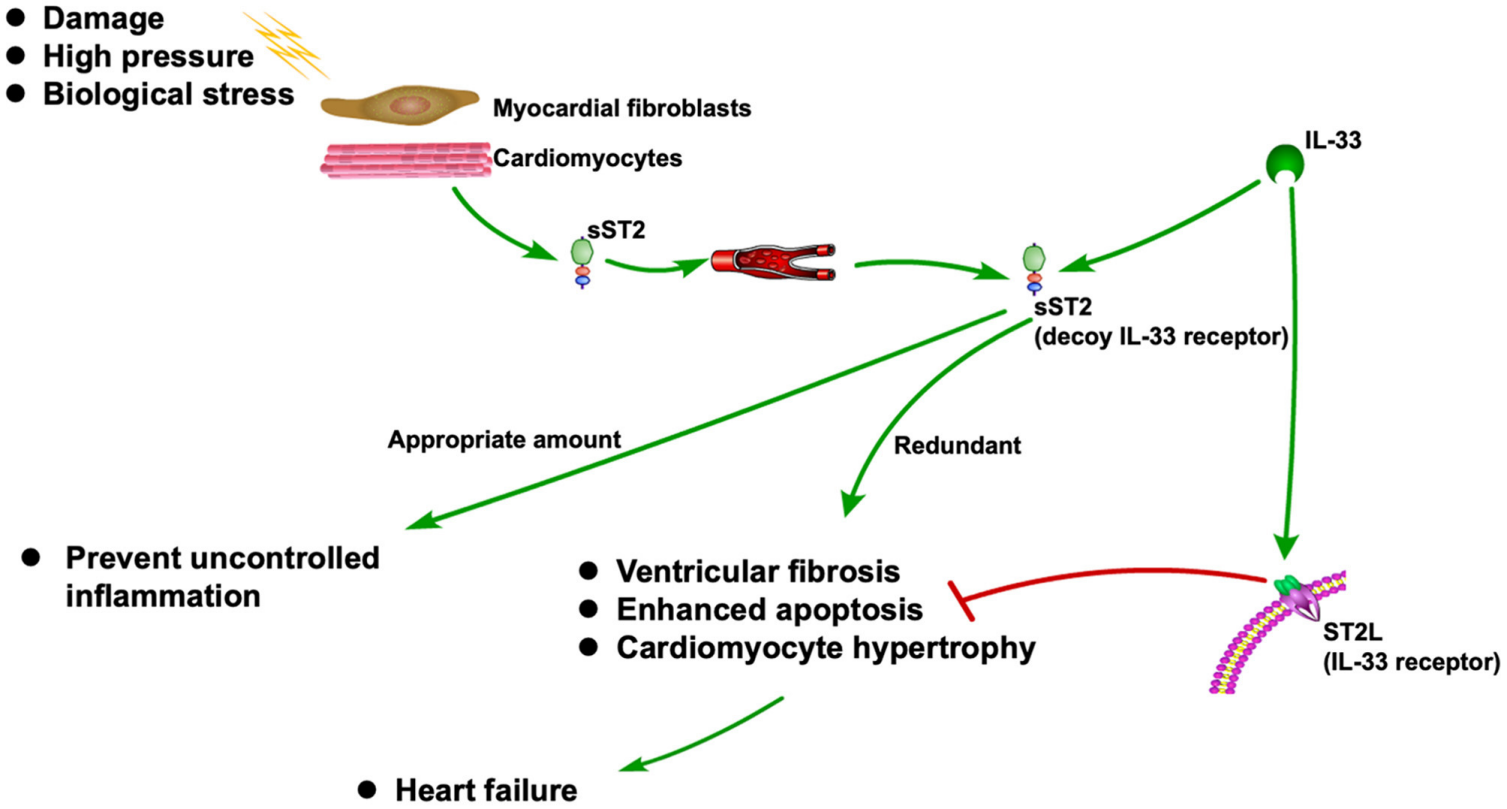
ST2 pathway in CVD.

sST2 is released when cardiomyocytes stretch, neutralizing its ligand IL-33 (13, 14). It is also associated with inflammation during the MI and HF (15). As a decoy receptor of IL-33 receptor (ST2L), an appropriate amount of sST2 can prevent uncontrolled inflammation. However, redundant sST2 could also block the advantageous biological effect of IL-33 due to its competitive role against ST2L, which will eventually cause HF (15). Recently, sST2 is frequently reported to be associated with CVDs, especially HF (16–18). The ST2 pathway in CVD is shown in Figure 1.

However, a meta-analysis of sST2 levels in different CVDs has not yet been conducted. Therefore, the present study aimed to investigate sST2 levels in patients with ischemic heart disease (IHD), MI, and HF and to explore the value of sST2 in the diagnosis and prognosis of CVD.

## Materials and Methods

### Literature Search Strategy

Two independent researchers comprehensively searched four online databases (PubMed, Embase, Cochrane, and Web of Science), with the latest search conducted on October 1, 2020. The keywords searched in PubMed included “IL1RL1 protein, human” combined with “myocardial ischemia”, “myocardial infarction”, and “heart failure”. The search strategy for PubMed was refined by using the following PubMed/MeSH terms: “IL1RL1 protein, human” [MeSH terms] or “ST2 protein, human” [abstract/title], and “Myocardial Ischemia” [MeSH terms], “Ischemic Heart Diseases” [abstract/title], “Myocardial Infarction” [MeSH terms], “Cardiovascular Stroke” [abstract/title], “Heart Failure” [MeSH terms], or “Cardiac Failure” [abstract/title]. In an effort to obtain maximum search results, we did not impose language restrictions during the search process.

### Inclusion Criteria

The inclusion criteria for studies in the systematic review and meta-analysis included (1) case–control studies; (2) studies comparing ST2 serum concentrations between healthy humans and patients with HF or cardiac diseases; (3) studies with sufficient data provided to obtain the weighted mean difference (WMD), 95% confidence interval (95% CI), or *p*-values for outcomes; and (4) studies with no significant differences in sex, age, diabetes, and hypertension observed between the control and disease groups.

### Exclusion Criteria

Studies were excluded if they contained any of the following exclusion criteria: (1) duplicated studies; (2) poor-quality studies; (3) uncontrolled studies or studies with control groups containing unhealthy samples; (4) studies not focusing on the diagnostic role of ST2 in HF or cardiac diseases; and (5) studies with insufficient data provided to obtain a WMD or 95% CI.

### Data Abstraction and Quality Assessment

Data from the included studies included the first author's name, publication year, sample characteristics (unit, size, method, and age), mean value, and standard deviation (SD). If the study data were not reported as mean value ± SD, researchers would independently estimate the mean and SD based on the sample size, medium, maximum or minimum values, range, and interquartile ranges, based on studies by Luo et al. (19) and Wan et al. (20).

Quality assessment of the included studies was conducted using the Newcastle–Ottawa Quality Assessment Scale (NOS), and studies with NOS scores ≥7 were considered of high quality. The assessment criteria were as follows:

1. selection: ① adequate definition of cases, ② representativeness of the cases, ③ selection of controls, and ④ definition of controls;
2. comparability: ⑤ comparability of cases and controls based on the design or analysis; and
3. exposure: ⑥ ascertainment of exposure, ⑦ same method of ascertainment for cases and controls, and ⑧ nonresponse rate.

### Statistical Analysis

The data were divided into three groups for statistical analysis: serum sST2 levels in IHD (group 1); serum sST2 levels in MI (group 2); and serum sST2 levels in HF (group 3). Within group 3, two subgroups were identified: serum sST2 levels in HF measured by ELISA (subgroup 3.1) and serum sST2 levels in HF measured using the Presage^®^ ST2 assay kit or commercially available immunoassays (subgroup 3.2).

Review Manager (RevMan) software version 5.3 (The Cochrane Collaboration, 2014) was used to perform the meta-analysis. WMD and 95% CI were used between groups. Heterogeneity was evaluated using Cochran's *Q* test and Higgins *I*^2^, and significant heterogeneity was shown when *p* < 0.05 and/or *I*^2^ > 50%. A random-effect model was applied if significant heterogeneity was determined; if not, a fixed-effect model was used.

### Receiver Operating Characteristic Curve

The mean sST2 concentration values for each study in group 3 and subgroup 3.2 were used to plot ROC curves using IBM SPSS Statistics version 26.0 software for Mac (IBM Corp., Armonk, NY, USA).

## Results

### Included Studies

**Figure 2:**
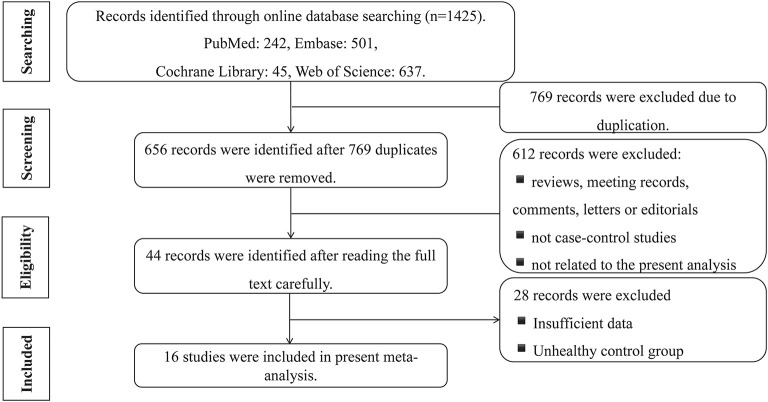
Flow diagram of literature search and exclusion process.

A total of 1,425 studies were searched, comprising 242 from PubMed, 501 from Embase, 45 from Cochrane Library, and 637 from Web of Science. A total of 769 studies were excluded because they duplicated other studies. After reading of the title and abstract, according to the exclusion criteria, 612 studies were removed because the studies were (a) reviews, meeting records, comments, letters, or editorials; (b) not case–control studies; and (c) not related to the present meta-analysis (focused on other diseases, the prognosis of sST2, etc.) Among the remaining 44 studies, two independent researchers read the full studies and excluded 28 records due to insufficient data or the inclusion of control groups with unhealthy individuals. Finally, 16 studies were included in the present meta-analysis (Figure 2).

### Characteristics of the Included Literature

All details of the 16 studies are presented in Table 1. Among the 16 studies, seven focused on IHD (21–23, 27, 28, 33, 35), among which four focused on MI (23, 27, 28, 35), and the others focused on HF. Four studies (30, 31, 34, 36) used a Presage^®^ ST2 assay kit to measure serum sST2 levels, while the remaining studies used ELISA or other immunoassays to complete this task. Most of the studies used weight/volume as the unit of measurement for serum sST2 levels, while Apple et al. used U/ml as the measurement unit (21). Therefore, in the meta-analysis of group 1, standard mean difference (SMD) was used to evaluate the results, thus eliminating the effect of different measurement units used in the various studies. WMD was used to evaluate the effect on the original measurement units for studies in groups 2 and 3. The studies were separated into three groups, as described above, and two subgroups in group 3 based on the method used to determine serum sST2 levels. The NOS scores of all 16 studies were ≥7, indicating that all included studies were of high quality (Table 2).

**Table 1:** The characteristics of the included studies.

**Author**	**Year**	**Unit**	***n***	**Analysis method**	**Disease**
Apple et al. (21)	2012	U/ml	464	a novel immunoassay	Cardiovascular disease
Aslan et al. (22)	2019	ng/ml	152	ELISA	Microvascular angina
Awada et al. (23)	2019	pg/ml	39	ELISA	Acute coronary syndrome
Cui et al. (24)	2018	pg/ml	247	ELISA	HFpEF, HFrEF
Emil et al. (8)	2019	μg/L	193	Presage^®^ ST2 assay kit	HFpEF, HFrEF
Fhaid et al. (25)	2019	pg/ml	60	ELISA	Congestive heart failure (LVEF <40%)
Firouzabadi et al. (26)	2020	pg/ml	66	ELISA	Heart failure (LVEF <50%, secondary to IHD)
Fu et al. (27)	2019	ng/L	110	ELISA	Acute myocardial infarction
Karimzadeh et al. (28)	2017	pg/ml	81	ELISA	Acute myocardial infarction
Luo et al. (29)	2017	μg/L	752	ELISA	HFpEF
Meijers et al. (30)	2016	ng/ml	111	Presage^®^ ST2 assay kit	Congestive heart failure (LVEF <45%)
Mueller et al. (31)	2015	ng/ml	37	Presage^®^ ST2 assay kit	Heart failure (without co-morbidity)
Pryds et al. (32)	2019	ng/ml	42	Commercially available immunoassays	Chronic ischemic heart failure
Quick et al. (33)	2015	ng/ml	134	ELISA	Coronary artery disease
Santhanakrishnan et al. (34)	2012	ng/ml	151	Presage^®^ ST2 assay kit	HFpEF, HFrEF
Schernthaner et al. (35)	2017	pg/ml	194	ELISA	STEMI, NSTEMI

*ST2, suppression of tumorigenicity 2; ELISA, enzyme-linked immunosorbent assay; HFpEF, heart failure with preserved ejection fraction; HFrEF, heart failure with reduced ejection fraction; STEMI, ST segment elevation myocardial infarction; NSTEMI, non-ST segment elevation myocardial infarction*.

**Table 2:** NOS score of included studies.

**Study**	**Quality indicators from the NOS**								
	**Selection**				**Outcome Assessment**	**Exposure**			**Scores**
	**①**	**②**	**③**	**④**	**⑤**	**⑥**	**⑦**	**⑧**	
Apple et al. (21)	*	*	*		**	*	*		7
Aslan et al. (22)	*	*	*	*	**	*	*		8
Awada et al. (23)	*	*	*	*	*	*	*		7
Cui et al. (24)	*	*	*	*	*	*	*		7
Emil et al. (8)	*	*	*	*	*	*	*		7
Fhaid et al. (25)	*	*	*	*	*	*	*		7
Firouzabadi et al. (26)	*	*	*	*	**	*	*		8
Fu et al. (27)	*	*	*	*	*	*	*		7
Karimzadeh et al. (28)	*	*	*	*	**	*	*		8
Luo et al. (29)	*	*		*	**	*	*		7
Meijers et al. (30)	*	*	*	*	*	*	*		7
Mueller et al. (31)	*	*	*	*	*	*	*		7
Pryds et al. (32)	*	*	*	*	**	*	*		8
Quick et al. (33)	*	*	*	*	*	*	*		7
Santhanakrishnan et al. (34)	*	*	*	*	*	*	*		7
Schernthaner et al. (35)	*	*	*	*	*	*	*		7

*The NOS evaluation included (1) selection: ① adequate definition of cases, ② representativeness of the cases, ③ selection of controls, and ④ definition of controls; (2) comparability: ⑤ comparability of cases and controls based on the design or analysis [(a) the study controls for cardiac disease and (b) the study controls for rate of sex, age, diabetes, and hypertension; the study that controlled both (a) and (b) was scored 2, but if only (a) was controlled, the study was scored 1]; and (3) exposure: ⑥ ascertainment of exposure, ⑦ same method of ascertainment for cases and controls, and ⑧ nonresponse rate*.

### Meta-Analysis

**Figure 3:**
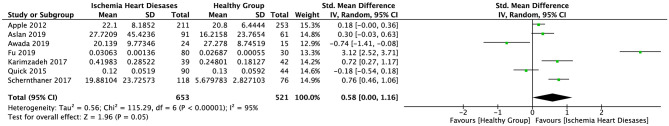
Forest plot of sST2 levels between IHD and healthy groups.

**Figure 4:**
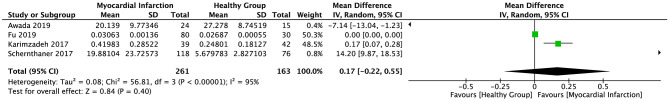
Forest plot of sST2 levels between MI and healthy groups.

**Figure 5:**
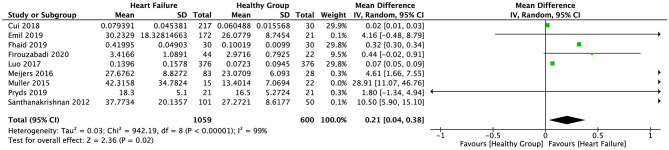
Forest plot of sST2 levels between HF and healthy groups.

**Figure 6:**
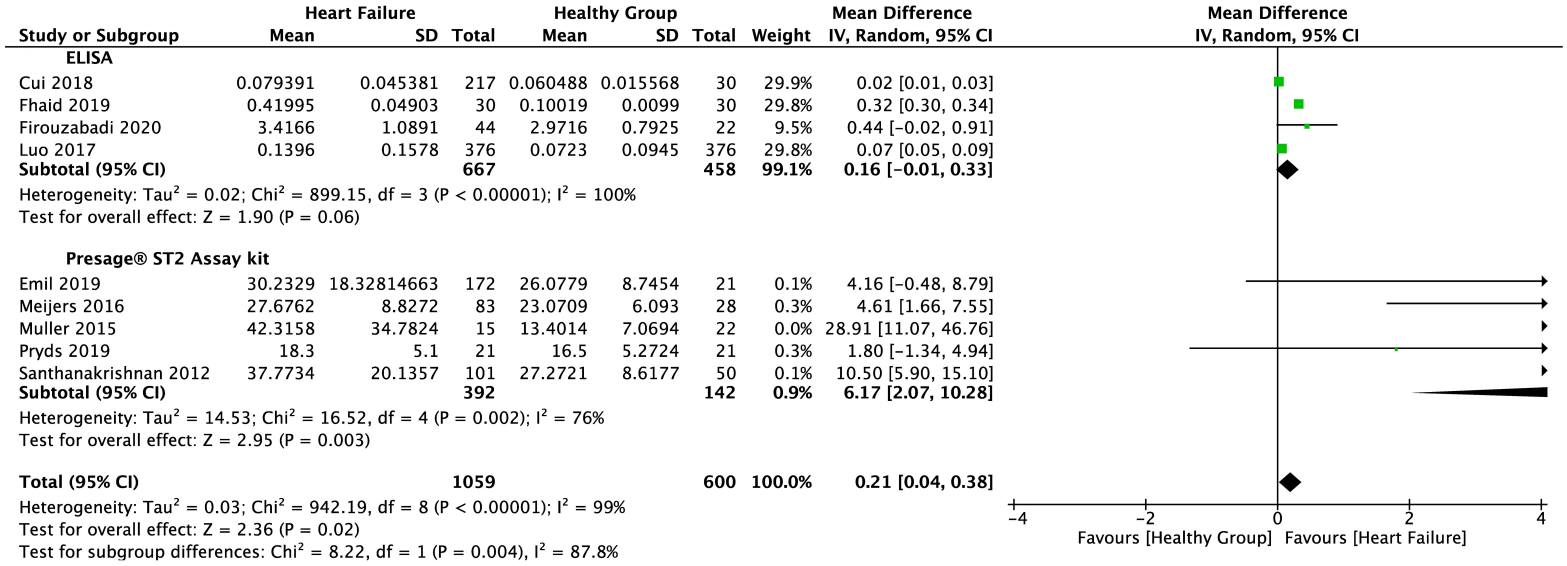
Forest plot of sST2 levels between HF and healthy subgroups with different assay methods (ELISA and Presage^®^ ST2 assay kit).

The random-effect model was applied to all three groups due to the presence of significant heterogeneity. No statistical difference was found in the serum sST2 levels between healthy individuals and IHD patients in group 1 (SMD = 0.58, 95% CI = 0.00–1.16, *p* = 0.05; *I*^2^ = 95%, *p* < 0.00001) (Figure 3) or MI patients in group 2 (WMD = 0.17, 95% CI = −0.22–0.55, *p* = 0.40; *I*^2^ = 95%, *p* < 0.00001) (Figure 4). However, a statistical difference was determined between the serum sST2 levels of HF patients and healthy individuals in group 3 (WMD = 0.21, 95% CI = 0.04–0.38, *p* = 0.02; *I*^2^ = 99%, *p* < 0.00001) (Figure 5). Interestingly, no statistical difference was found in subgroup 3.1 (WMD = 0.16, 95% CI = −0.01–0.33, *p* = 0.06; *I*^2^ = 100%, *p* < 0.00001), whereas a statistical difference was determined in subgroup 3.2 (WMD = 6.17, 95% CI = 2.07–10.28, *p* = 0.003; *I*^2^ = 76%, *p* = 0.002) (Figure 6).

### ROC Curves

**Figure 7:**
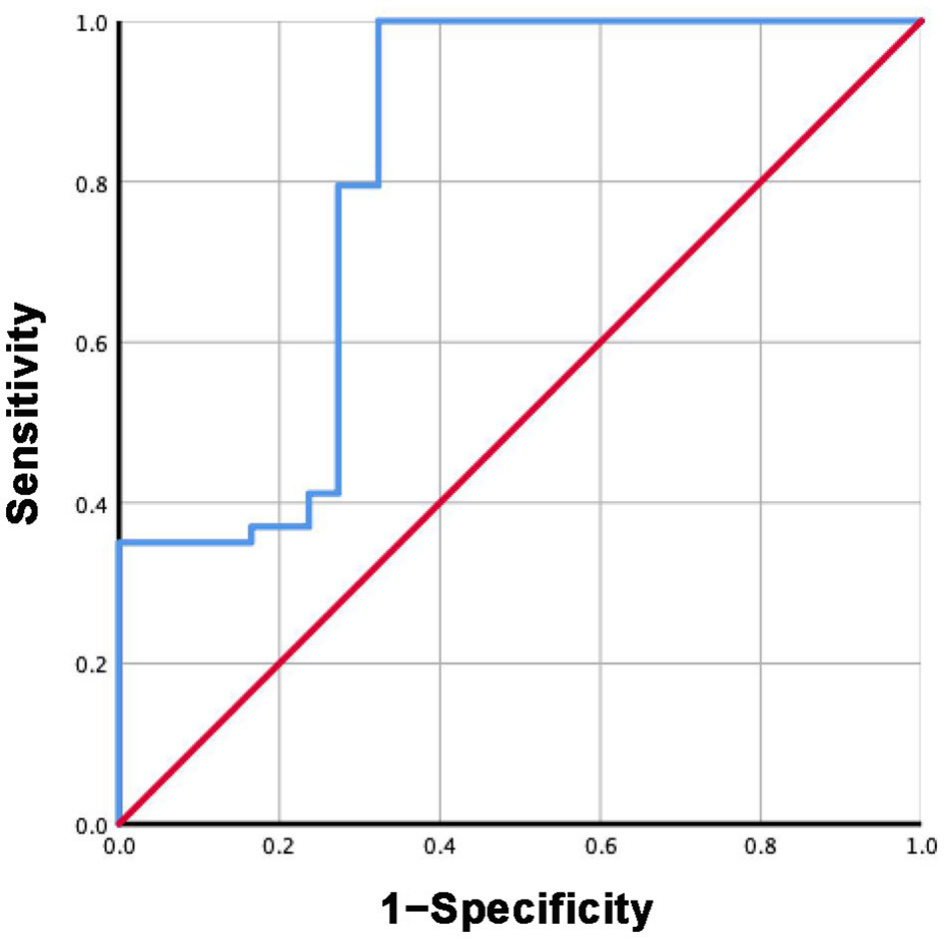
ROC curve of sST2 levels between HF and healthy groups.

**Figure 8:**
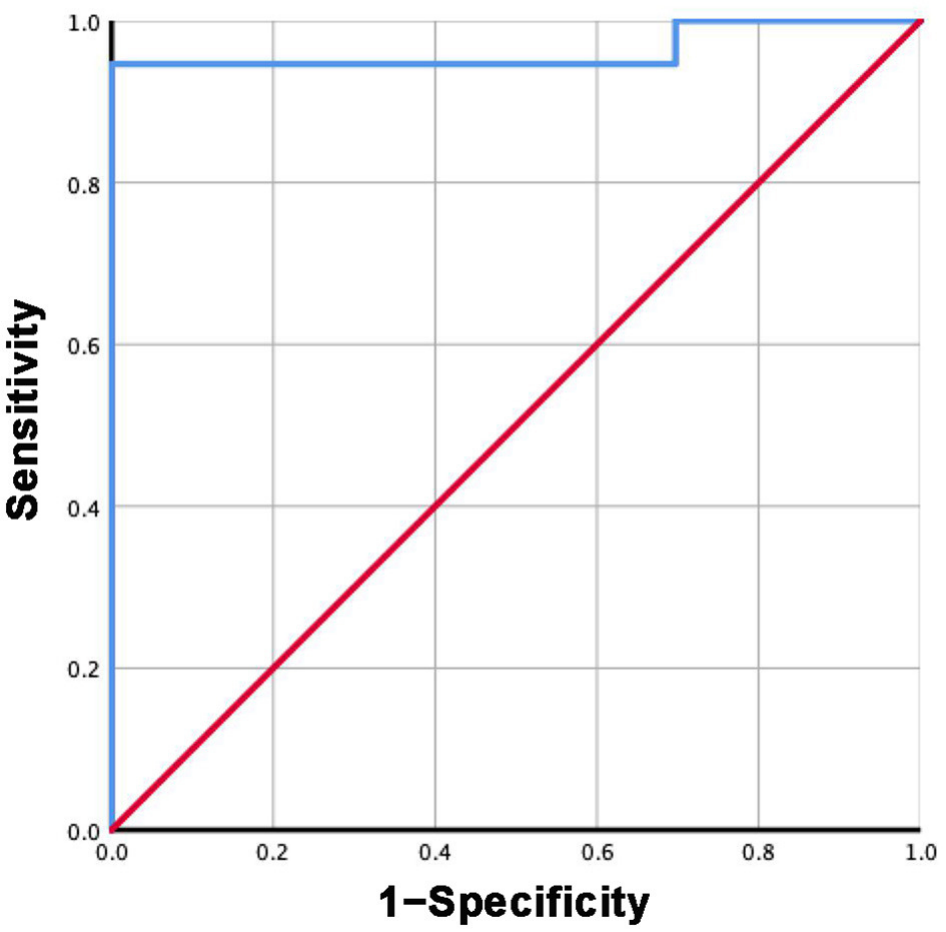
ROC curve of sST2 levels between HF and healthy groups measured by Presage^®^ ST2 assay kit.

Based on the mean sST2 concentrations reported in each study in group 3 and subgroup 3.2, the specificity and sensitivity of ST2 were calculated. The ROC curves are shown in Figures 7, 8. For group 3, the area under the curve (AUC) is 0.816, *p* = 0.000, 95% CI = 0.792–0.840. For subgroup 3.2, the AUC is 0.963, *p* = 0.000, 95% CI = 0.947–0.979, with a cutoff value of 27.4742 ng/ml when the sensitivity was 0.946 and the specificity was 1.000.

## Discussion

Biomarkers have made a significant contribution to the diagnosis and prognosis of disease. Although strongly associated with inflammatory and autoimmune diseases, ST2 has also been found to play a role in the diagnosis and prognosis of CVD (11, 36). As one of the isoforms of ST2, sST2 has recently become a promising prognostic indicator for patients diagnosed with HF and a useful tool for risk stratification (37). Due to its prognostic value, sST2 was recommended by the American College of Cardiology Foundation (ACC)/American Heart Association (AHA) as an important biomarker for monitoring HF patients in 2013 (38).

To clearly distinguish sST2 levels in various CVDs, the present study specifically analyzed sST2 levels in patients with IHD, MI, and HF. Among the included studies, seven focused on IHD, which is a disease in which cardiomyocytes do not receive sufficient blood and oxygen supply. Four of these seven studies focused on MI, in which irreversible necrosis of cardiomyocytes occurs as a result of severe ischemia. To investigate sST2 levels in MI patients, we performed a separate meta-analysis of these four studies (group 2).

The meta-analysis findings indicated that serum sST2 levels did not differ significantly in patients with IHD or MI compared with healthy individuals but were remarkably increased in patients with HF. Herein, the biological character of the ST2 molecule; its involvement in heart disease pathophysiology; the diagnostic, prognostic, and treatment values of ST2; and the limitations of the study will be discussed in order to support the application of this biomarker in a clinical setting.

### Molecular Biological Characteristic of ST2

ST2 is a member of the Toll-like/IL-1 receptor (TIR) superfamily, the members of which are defined by a common intracellular domain, the TIR domain (32). The functional ligand of ST2 is IL-33. sST2 can also directly bind to IL-33 and act as a decoy receptor, inhibiting membrane-bound ST2 and subsequent signal transduction (22). sST2 is mainly produced by myocardial fibroblasts and stressed or injured cardiomyocytes and is also expressed in mast cells, epithelial cells, smooth muscle cells, and endothelial cells from the cardiac and microvascular systems (39, 40).

The Framingham Heart Study found that age and sex are important determinants of normal sST2 levels (41). Specifically, sex has a potentially crucial influence on sST2, in that female sST2 levels are lower than those of males at corresponding ages (42), which may be influenced by sex hormones (43). In addition, sST2 secretion follows a diurnal rhythm, with lower values in the morning, a peak in the afternoon, and lowest levels at night (43). Understanding the diurnal rhythm and sex differences affecting sST2 expression can help avoid false negatives in prognostic prediction.

Therefore, based on the above characteristics of sST2, relevant inclusion criteria were determined to avoid statistical differences in age and sex between the disease and control groups during the literature screening process. Meanwhile, the exclusion of studies with unhealthy individuals in the control group was strictly followed.

### sST2 Involvement in Heart Disease Pathophysiology

#### Cardiomyocyte Hypertrophy and Cardiac Fibrosis

Studies have shown that the IL-33/ST2 signaling pathway is a biomechanical activation system closely associated with cardiomyocyte hypertrophy and cardiac fibrosis (44). When cardiomyocytes stretch, sST2 is released, neutralizing its ligand IL-33, which is a key component in preventing myocardial fibrosis and hypertrophy (13, 14). Any change in the geometry or load conditions of the heart, such as MI, hypertension, and valvular heart disease, may change the mechanical strain imposed on a single cardiomyocyte, leading to cardiomyocyte hypertrophy, enhanced extracellular protein deposition (ventricular fibrosis), and eventually HF (45–47). As mentioned above, sST2 is positively correlated with cardiomyocyte hypertrophy and cardiac fibrosis. The increased wall thickness in hypertension tends to diminish wall stress and decrease oxygen demand. Cardiomyocyte hypertrophy is the most important of the pathophysiological changes in the heart, eventually contributing to ventricular wall thickening and stiffening (48). Therefore, both cardiomyocyte hypertrophy and cardiac fibrosis contribute to elevated serum sST2 levels in HF patients, which was corroborated by the current findings.

According to the present meta-analysis, no statistical differences in serum sST2 levels were found between the MI and IHD groups compared with the corresponding control groups. Remodeling occurs after MI, but expansion of the infarct zone is only found within 72 h after infarction. Beyond 72 h, remodeling via myocyte hypertrophy and alterations in ventricular architecture begin to affect post-infarction repair (49). The studies investigating MI and IHD included in the meta-analysis reported that blood samples were taken as soon as possible when patients were sent to the emergency room. Thus, increased sST2 levels may not have been observed within the hyperacute or acute phase of MI.

#### Inflammatory Processes

ST2L is a membrane-bound receptor of IL-33 located on the cardiomyocyte surface (50). Fibroblasts release IL-33, which prevents cardiomyocyte apoptosis and improves cardiac function through ST2 signaling in the myocardium under conditions of damage, high pressure, or biological stress (51). Even if sST2 protects against overload due to inflammatory injury, it could also block the advantageous biological effect of IL-33 because sST2 acts as a competitive inhibitor of ST2L (15). Therefore, high sST2 concentrations enhance apoptosis, cardiomyocyte hypertrophy, and cardiac fibrosis, which eventually cause irreversible damage after MI, leading to HF.

We speculate that higher serum sST2 concentrations after MI may cause a worse prognosis because sST2 blocks the beneficial effects of IL-33, such as reducing fibrosis and hypertrophy, preventing apoptosis, preserving ventricular function, and improving survival (52). Therefore, sST2 may be used to evaluate risk of death after MI.

### Clinical Application

#### Diagnosis

As early as 2013, sST2 was recommended by ACC/AHA as a predictor of hospitalization and death in patients with chronic HF, in addition to BNP (38). The stability of sST2 (48 h at room temperature, 7 days at 4°C, and 2 months at −20°C or −80°C) reflects its suitability for clinical laboratory analysis (42). Some studies have reported an upper reference of 35 ng/ml for sST2 (48), which is higher than the cutoff value found in the current study (27.4742 ng/ml). According to the AUC values obtained in the present meta-analysis, the potential diagnostic value of sST2 in HF is considerable. However, as mentioned above, sex and diurnal rhythm (41–43) should be considered when setting the cutoff value for diagnosis during clinical application.

Moreover, some studies have reported that serum sST2 levels may be higher after MI, especially after 3–5 days (28, 34). However, serum sST2 levels did not differ significantly between patients with MI and healthy individuals in the present meta-analysis. Based on the pathophysiology of sST2 in the CVDs mentioned above, we speculate that sST2 levels might be elevated after the acute phase of MI, which warrants further confirmation. Other CVDs, such as hypertension and valvular heart disease, could also lead to higher sST2 levels; thus, differential diagnoses should be distinguished based on the sST2 level. In addition, elevated sST2 levels have been observed in multiple non-cardiac diseases, such as inflammation, asthma, fibroproliferative diseases, rheumatoid arthritis, autoimmune diseases, sepsis, and trauma (41, 53). Consequently, the diagnosis of HF requires comprehensive assessment. BNP (or NT-pro BNP) is a classic biomarker for the diagnosis of HF. Previous studies have reported that sST2 has a weaker predictive value than NT-proBNP in the diagnosis of HF (54, 55). However, BNP (or NT-pro BNP) has demonstrated weaker differentiating ability in HF and renal failure, while sST2 is hardly influenced by kidney function (56–58). Therefore, sST2 should be used together with BNP (or NT-pro BNP) as an auxiliary diagnostic marker to diagnose HF.

Interestingly, a statistical difference was found between HF patients and healthy individuals when serum sST2 levels were determined using different methods (Presage^®^ ST2 assay kit versus ELISA). We speculate that different operational procedures employed to perform ELISA may cause large heterogeneity and bias, which may decrease the statistical power of meta-analysis. Use of a commercial kit, like the Presage^®^ ST2 assay kit, eliminates differences in operational flow or reagents, enabling repeatable determination of sST2, thus minimizing heterogeneity and bias between laboratories and experiments. Therefore, we recommend that a commercial kit, such as Presage^®^ ST2 assay kit, be employed to evaluate sST2 levels in both research and clinical settings. Furthermore, although we provided a cutoff value of 27.4742 ng/ml, based on the Presage^®^ ST2 assay kit method, this value is only a preliminary value as only five studies were included in subgroup 3.2 and the original data of each individual was not accessible. Thus, a more accurate cutoff value should be investigated in future studies with larger sample sizes.

#### Prognosis

Even if no diagnostic value had been found in the present meta-analysis, the prognostic value of sST2 in MI and IHD has been clarified. In patients with AMI, elevated ST2 levels are often associated with poor prognosis (59). Measurement of sST2 early after AMI predicts left ventricle (LV) function and recovery after AMI, which may interlink the RAAS and IL-33/sST2 pathways (60). The prognostic value of sST2 in AMI patients has been demonstrated, but its pathogenesis requires further research.

For HF, sST2 has not only diagnostic but also prognostic value. In HF patients classified as chronic New York Heart Association (NYHA) classes III to IV, sST2 is an independent predictor of subsequent mortality (61). Some studies have demonstrated that sST2 levels have predictive value for mortality in HF patients in both the short and long term (62, 63). A higher sST2 concentration (upper reference limit of 35 ng/ml) provides a substantial predictive risk of cardiovascular events, such as progressive LV failure, hospitalization for HF, and death (64). It is worth noting that measuring sST2 combined with BNP or NT-proBNP is better than merely measuring NT-proBNP, because sST2 has a better predictive value than NT-proBNP in myocardial fibrosis and remodeling, which is a known indicator of HF severity (65, 66). Wu et al. also reported that the low biological variability of sST2 compared to BNP or NT-proBNP enables serial monitoring of sST2, which is preferred over single measurement (65).

The predictive role of sST2 in HF is somewhat limited in some situations. A study investigating cardiac resynchronization therapy (CRT) and markers and response to CRT (MARC) (67) reported that sST2 did not reflect changes in HF and LV ejection fluid (LVEF) before and after CRT. Further research is required to explore this phenomenon.

#### Treatment

Studies have shown that increased IL-33/ST2 signaling may induce heart protective effects, and MyD88 may represent a common point of ST2L and Toll-like receptor 4 (TLR-4) heart protection signaling. The following strategies can be applied: direct administration or enhanced IL-33 release from cardiac fibroblasts, administration of ST2L agonists or sST2 antagonists, or administration of an adaptor protein numerator (68–70). Unique cardiovascular targets may be discovered if precise cytoplasmic and nuclear effects can be elucidated. Experiments have shown that a short course of IL-33/ST2 can prevent cardiomyocyte apoptosis and improve the results of experimental MI without lung inflammation (53). Therefore, transient IL-33 treatment may improve cardiovascular outcomes and minimize adverse inflammatory reactions.

### Limitations

Among the 16 studies included in the current meta-analysis, four measurement methods were used to evaluate serum sST2 levels: the Presage^®^ ST2 assay kit, ELISA, commercially available immunoassays, and a novel immunoassay. Some researchers have reported that different methods may cause up to a 100-fold difference in results. Further, although the Presage^®^ ST2 assay kit has been cleared by the U.S. Food and Drug Administration and has received the Conformitè Europèenne (CE) mark, ELISA remains a research-only method (42). Different methods yield different results, which may cause significant heterogeneity and weaken the statistical power of the analysis. This is one of the limitations of the present meta-analysis. Use of a commercial kit, such as the Presage^®^ ST2 assay kit, would standardize the methodology and enable accurate comparisons between studies. Furthermore, one study used U/L as the measurement unit, which cannot be converted into ng/ml. SMD was used for this analysis, which weakens the value of the results for application in clinical diagnosis. Therefore, we suggest that researchers and doctors use the Presage^®^ ST2 assay kit to evaluate the level of sST2 on both bench and bedside.

Although we provided a cutoff sST2 value at the optimal sensitivity/specificity, the sample and study sizes were small, which is also one of the limitations of the present study. Meanwhile, inaccessibility of the original data from each individual sST2 level may also have led to the same problem. The present analysis is thus a preliminary investigation on the topic; more studies and original data are required to determine a precise cutoff value for sST2.

Finally, only four and seven studies were included in the MI and IHD groups, respectively. The resulting error and bias cannot be ignored during the statistical analysis and discussion of the results. Meanwhile, *p* = 0.05 was applied in group 1, but we believe a statistical difference exists when *p* < 0.05. In both groups 1 and 2, the random-effect model was employed because high heterogeneity existed, which reduced the statistical power of the results. These factors may have caused a bias in the meta-analysis results. Therefore, more research assessing the diagnostic value of sST2 in MI and IHD is needed, which will lead to more accurate results than those presented here.

## Conclusion

The results of the present meta-analysis of serum sST2 levels in different CVDs demonstrate that serum sST2 levels in HF patients are remarkably higher than those in healthy individuals. However, serum sST2 levels did not differ significantly between IHD or MI patients and healthy individuals. Therefore, we believe that ST2 can be used as an auxiliary diagnostic biomarker of HF.

## Data Availability Statement

The original contributions presented in the study are included in the article/supplementary material, further inquiries can be directed to the corresponding author/s.

## Author Contributions

ZC contributed to the conception of the study. TZ and CX performed the meta-analyses and wrote the manuscript. RZ edited and revised the original manuscript. All authors contributed to the article and approved the submitted version.

## Conflict of Interest

The authors declare that the research was conducted in the absence of any commercial or financial relationships that could be construed as a potential conflict of interest.

## Publisher's Note

All claims expressed in this article are solely those of the authors and do not necessarily represent those of their affiliated organizations, or those of the publisher, the editors and the reviewers. Any product that may be evaluated in this article, or claim that may be made by its manufacturer, is not guaranteed or endorsed by the publisher.
